# How good are my data and what is the resolution?

**DOI:** 10.1107/S0907444913000061

**Published:** 2013-06-13

**Authors:** Philip R. Evans, Garib N. Murshudov

**Affiliations:** a MRC Laboratory of Molecular Biology, Hills Road, Cambridge CB2 0QH, England

**Keywords:** data reduction, data scaling, software, data statistics

## Abstract

Following integration of the observed diffraction spots, the process of ‘data reduction’ initially aims to determine the point-group symmetry of the data and the likely space group. This can be performed with the program *POINTLESS*. The scaling program then puts all the measurements on a common scale, averages measurements of symmetry-related reflections (using the symmetry determined previously) and produces many statistics that provide the first important measures of data quality. A new scaling program, *AIMLESS*, implements scaling models similar to those in *SCALA* but adds some additional analyses. From the analyses, a number of decisions can be made about the quality of the data and whether some measurements should be discarded. The effective ‘resolution’ of a data set is a difficult and possibly contentious question (particularly with referees of papers) and this is discussed in the light of tests comparing the data-processing statistics with trials of refinement against observed and simulated data, and automated model-building and comparison of maps calculated with different resolution limits. These trials show that adding weak high-resolution data beyond the commonly used limits may make some improvement and does no harm.

## Abstract 2

The new scaling program *AIMLESS* is described and tests of refinements at different resolutions are compared with analyses from the scaling step.

## 1. Introduction

Following integration of the spots on a set of X-ray diffraction images to produce a list of reflection intensities, a series of operations are performed on the data, usually referred to as ‘data reduction’. These processes include determination of the point group and if possible the space group, checking for consistent indexing where there are alternatives, putting all of the data on a common scale, deciding whether to reject parts of the collected data or to cut the resolution and estimating the structure amplitude |*F*| from intensity. Statistics on the internal consistency of the data also provide a good indication of the overall quality of the data set. Algorithms and methods for data reduction have been well documented in many papers (*e.g.* Fox & Holmes, 1966 ▶; Otwinowski *et al.*, 2003 ▶; Evans, 2006 ▶, 2011 ▶; Kabsch, 2010 ▶) and the details will not be repeated here. Scaling attempts to correct for contributions to the measured intensities arising from experimental conditions which vary during data collection, such as variations in the incident-beam intensity, the volume of the crystal illuminated, absorption in the primary or secondary beam and average radiation damage. This is performed by trying to make all symmetry-related or replicated measurements of a reflection intensity equal, *i.e.* to make the data as internally consistent as possible.

This paper describes a new data-scaling program, *AIMLESS*, and discusses criteria for deciding the ‘resolution’ of a measured data set. In the *CCP*4 context (Winn *et al.*, 2011 ▶), *AIMLESS* is used immediately after the program *POINTLESS*, which determines the likely point group and possible space group, as well as optionally combining data from multiple files each containing a ‘sweep’ of data and putting them on a common indexing system if necessary (Evans, 2011 ▶).^1^ *AIMLESS* is then followed by *CTRUNCATE*, which calculates the structure amplitudes |*F*| from the intensities and outputs various intensity statistics mainly to detect twinning.

## 2. The scaling program *AIMLESS*

*AIMLESS* is a new implementation in C++ of a classic scaling method, designed to make it easy to add new scaling models and algorithms. It is a replacement for the earlier *CCP*4 program *SCALA* (Evans, 2006 ▶, 2011 ▶) and at present uses a very similar scale model. The function minimized is
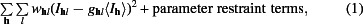
where *I*_**h***l*_ is the *l*th observation of reflection **h**, *g*_**h***l*_ is its associated inverse scale, *w*_**h***l*_ = 1/σ^2^(*I*_**h***l*_) and 〈*I*_**h**_〉 is the weighted average intensity for all observations *l* of reflection **h** or its symmetry mates. The inverse scale *g*_**h***l*_ comes from the refined scale model and is a function of the crystal rotation angle ϕ as a proxy for primary beam direction **s**_1_ and for radiation dose (or time) and the secondary beam direction **s**_2_: *g* = *g*_primary_(ϕ)exp[−2*B*(ϕ)sin^2^θ/λ^2^]*g*_secondary_(**s**_2_). The relative *B*-­factor term is largely an average radiation-damage correction. The smoothed primary scale factors *g*_primary_(ϕ) and relative *B* factors *B*(ϕ) are determined at suitable intervals in ϕ and are interpolated using Gaussian weights. The secondary beam correction *g*_secondary_(**s**_2_) is determined as a sum of spherical harmonic terms. Parameter restraint terms (ties) include a sphericity restraint on *g*_secondary_(**s**_2_), tying all spheri­cal harmonic coefficients to zero and optional ties between adjacent primary scales and relative *B* factors.

*AIMLESS* iterates the scaling step with an optimization of the standard error estimates on each observation σ(*I*_**h***l*_) and outlier rejection. Note that the scaling process is generally hugely overdetermined (many more observations than parameters, *e.g.* 45 000 observations for 30 parameters) and that weak intensities do not contain much useful information about the scales, so that the scaling can be made faster (roughly linearly) by working with a selected subset of strong reflections. The principal steps in the process (in the current version) are as follows.

(i) Read all observations into a reflection-list object (*SCALA* does not store the observations but rereads them for each scaling cycle *etc.*, as storing large data sets was impracticable when *SCALA* was written). Sort symmetry-related observations and partial observations together if necessary.
(ii) Set up the scaling model depending on what data are present and explicit user-given control instructions (if any). For the smoothly varying scale and *B* factors, decide on suitable interval for the scales for each ‘run’ or ‘sweep’ of contiguous images, depending on the total length of the sweep and default or specified values.
(iii) Obtain initial rough scale estimates from average intensities.
(iv) First-round scaling with a sample of a few thousand strong reflections with *I*/σ(*I*) greater than a suitable minimum value. At this stage the σ(*I*) estimates read from the integration program may not be very accurate but are good enough for this selection.
(v) First outlier rejection, using an algorithm similar to that described in §A5 of Evans (2006 ▶).
(vi) For data from *MOSFLM*, which outputs two estimates of each intensity, optimize the level of intensity *I*_mid_ at which to switch (smoothly) between using the profile-fitted value *I*_prf_ for weak data and summation integration *I*_sum_ for strong data, 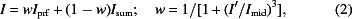
where *I*′ is the summation integration intensity before application of the Lorentz and polarization corrections. The exact form of this weighted mean is not critical, as ideally the two estimates are the same.
(vii) First optimization of σ(*I*) estimates (see §[Sec sec2.1]2.1).
(viii) Main scaling with strong reflections chosen on normalized intensities *E* ^2^ (typically choosing only observations with 0.8 < *E* ^2^ < 5). This gives a subset of data distributed over all resolution ranges.
(ix) Second outlier rejection as before.
(x) Final optimization of σ(*I*) estimates as before.
(xi) Final outlier rejection as before.
(xii) Accumulate and print statistics.
(xiii) Output merged or unmerged reflection lists to files.

### 2.1. Standard error estimates

Initial estimates of the standard error of each intensity observation, σ(*I*_**h***l*_), are generally underestimated by all integration programs, so *AIMLESS*, like *SCALA*, updates the σ(*I*) estimates in an attempt to make the average standard error match the average scatter of observations as a function of intensity only. The mismatch arises partly from the unknown ‘gain’ of the detector (*i.e.* detector units per photon), which scales the Poisson-statistic error estimates (as well as correcting for the detector point-spread function), and partly from a variety of instrumental instabilities which cause an increase in error with increasing intensity. If the error estimate were correct in explaining the observed discrepancies within the data set, then the normalized deviations
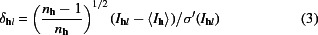
(where *n*_**h**_ is the number of observations of reflection **h** and here *I*_**h***l*_ is the scale-corrected value, *i.e.* *I*_**h***l*_/*g*_**h***l*_) should be distributed with a mean of 0.0 and a standard deviation of 1.0.^2^ The standard error estimate is then adjusted to
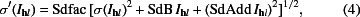
optimizing the values of the ‘correction’ factors SdFac, SdB and SdAdd to make variance(δ_**h***l*_) equal to 1.0 over all intensity ranges. The value of the term inside the square root is (arbitrarily) set to a minimum value of 0.1σ(*I_**h***l*_*)^2^ to avoid possible negative values (a rare possibility). A separate set of correction factors is determined for each ‘run’ (usually, although an option is available to use the same value for all runs) and separately for fully recorded and partially recorded observations (if relevant). This is performed by minimizing 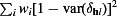 summed over all intensity ranges *i*, currently with equal weights *w*_*i*_ on each intensity bin and loose restraints on SdB parameters to avoid extreme values. Final values of variance(δ_**h***l*_) as a function of intensity for each run are plotted in the output of the program as an indication of the success (or otherwise) of this ‘correction’. Sdfac can be identified primarily with the uncertainty in the detector gain and Sdadd with general instability factors which lead to an error proportional to intensity (see, for example, Diederichs, 2010 ▶), but SbB has no obvious physical interpretation. However, this factor helps to flatten the plot of variance(δ_**h***l*_) against intensity and may be justified as an empirical ‘correction’ factor. It should be noted that this ‘correction’ to the error estimates is a fairly crude approximation, as it assumes there are no major residual systematic errors (such as those arising from radiation damage, for example) and that the correction can be parameterized purely on intensity.

## 3. Analysis of data quality

Once we have a set of scale factors to put all the intensities on a common scale and improved estimates of the error on each intensity, then we can analyse the data for internal consistency and for signal-to-noise ratio. We can do this as a function of image number (equivalent to time or crystal rotation) to detect radiation damage and weak or inconsistent regions of data and against resolution to decide on a high-resolution cutoff (see §4). Note that internal consistency measures are likely to underestimate the true error, since symmetry-related observations may suffer from the same systematic error.

Internal consistency has traditionally been measured by *R* factors relating an individual observation *I*_**h***l*_ (after scaling) to the (weighted) average of all symmetry-related or replicate observations of the unique reflection **h**, 〈*I*_**h**_〉. The multiplicity-weighted *R*_meas_ is an improvement over *R*_merge_, as it is relatively insensitive to data multiplicity (Diederichs & Karplus, 1997 ▶; Weiss & Hilgenfeld, 1997 ▶; Weiss, 2001 ▶), whereas *R*_merge_ tends to increase with increasing multiplicity, even though the averaged intensities are improving. *R*_p.i.m._ provides an estimate of data quality after merging multiple observations.
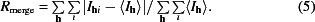


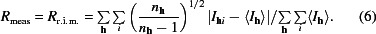


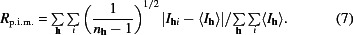



An alternative way of measuring internal consistency for the analysis against resolution is to split the observations randomly into halves and then calculate the linear correlation coefficient between the halves. This is arguably the most reliable measure and is discussed further below.

### 3.1. Analysis against ‘batch’ or image number

**Figure 1:**
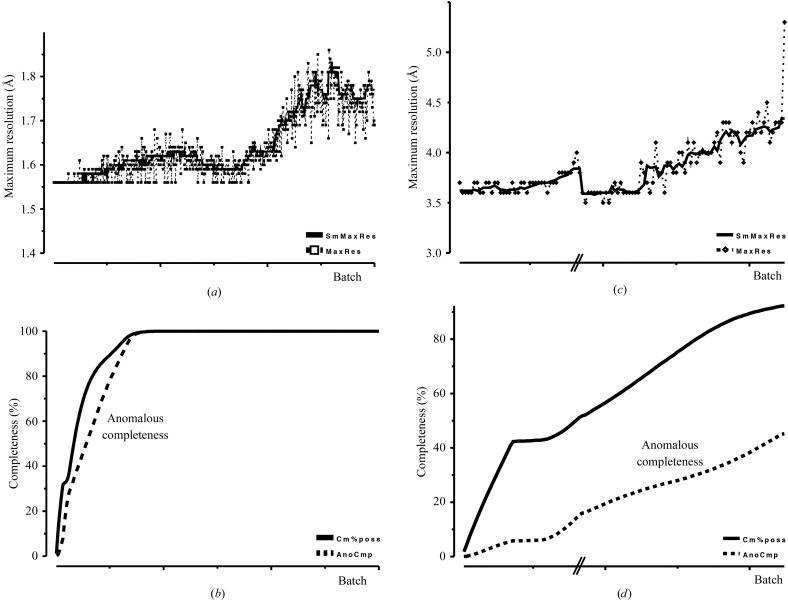
New graphs from *AIMLESS* against ‘batch’ or image number. (*a*, *c*) Nominal resolution estimated as the point at which 〈*I*/σ(*I*)〉 falls below 1.0, showing a trend to lower resolution with increasing radiation damage, with both values for individual batches and values smoothed over a 5° range. (*b*, *d*) Cumulative completeness for all data and anomalous differences. (*a*) and (*b*) show that in this good case the damaged data in the last third of the sweep can be safely discarded without reducing the completeness. (*c*) and (*d*) show graphs for a poor and incomplete data set from two crystals. At the end of this data collection the anomalous data are still very incomplete. Breaks in the *x* axis separate the two crystals.

Analysis of various parameters as a function of batch or image number, a proxy for crystal rotation, time or radiation dose, is useful to determine whether the crystal has suffered unduly from radiation damage or whether there are any other parts of the data which should be discarded. *AIMLESS* plots similar graphs of *R*_merge_ and of scale and relative *B* factors to those produced by *SCALA* (see, for example, Fig. 2 of Evans, 2011 ▶). Increasing negative values of the relative *B* factor are an indicator of deterioration with dose, although the *B* factor is also affected by factors other than radiation damage. *AIMLESS* adds two new plots against batch number: a rough estimate of the maximum resolution for each image and a cumulative completeness (for all data and anomalous pairs; see Fig. 1 ▶). The ‘maximum resolution’ is estimated from the point at which 〈*I*/σ(*I*)〉 falls below 1.0 for each batch: note that this 〈*I*/σ(*I*)〉 is without averaging multiple measurements (which would not generally occur on the same image), so will be smaller than the 〈*I*/σ〉 after averaging (§[Sec sec3.2.1]3.2.1). The plot shows the noisy values for each batch, as well as a smoothed plot typically averaged over a 5° range. This is only a rough estimate of resolution (see §[Sec sec4]4), but serves to illustrate any trends. The cumulative completeness plot helps in deciding whether cutting back data from the end because of radiation damage would compromise the completeness. Such decisions are more complicated if the data have been measured from multiple ‘sweeps’ or multiple crystals.

### 3.2. Analysis against resolution

In order to estimate the useful ‘resolution’ of the data, *i.e.* the resolution at which the data may be truncated without losing significant information, *AIMLESS* plots various measures of signal to noise and internal consistency against resolution. There is at present no general consensus on the optimum criteria for interpretation of these plots and how to estimate the point at which adding additional high-resolution data is not adding anything useful: the true ‘resolution’ of a data set has often been a point of contention with referees of papers.

#### 3.2.1. Signal-to-noise ratio

One obvious way to judge data significance is from the average signal-to-noise ratio of the merged intensities as a function of resolution. This is calculated as
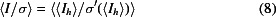
[after ‘correcting’ the σ′(*I*_**h***l*_) estimates; §[Sec sec2.1]2.1], *i.e.* for each reflection **h** the average intensity over symmetry mates 〈*I*_**h**_〉 is divided by its estimated error σ(〈*I*_**h**_〉) and this ratio is averaged in resolution bins [reported as Mn(I/sd) in the program output]. Commonly used resolution-cutoff levels are typically in the range 1–2: even in a resolution bin with 〈*I*/σ〉 = 1 a proportion of intensities are significantly above the noise level [∼5–7% *I* > 3σ, ∼20–25% negative]. 〈*I*/σ〉 is a good criterion for resolution cutoff, but it does suffer from uncertainties in the estimation of σ(*I*), both from inadequacies in the integration program and the necessary σ(*I*) ‘correction’ (see §[Sec sec2.1]2.1; Ian Tickle, in a private communication, has pointed out that the major correction applies to large intensities and therefore would not affect the weak high-resolution data relevant to determination of resolution cutoff). It should be noted that 〈*I*/σ〉 is not independent of measures of internal consistency because the corrections to σ′(*I*) are adjusted to match the scatter of observations. Thus, σ′(*I*) estimates are still likely to be underestimates of the true standard deviations.

#### 3.2.2. Measures of internal consistency

The traditional *R* factors measuring internal consistency, *R*_merge_ or better *R*_meas_ (§[Sec sec3]3), are not suitable measures for setting a resolution cutoff (Evans, 2011 ▶; Karplus & Diederichs, 2012 ▶), despite their apparent popularity with referees. As pointed out by Karplus and Diederichs, these *R* factors cannot be compared with the *R* factors in refinement, since *R*_merge_ and *R*_meas_ both tend to infinity as the data become weaker, while *R*_cryst_ (either *R*_work_ or *R*_free_) tends to a constant (see Appendix *A*1[App appa] and Luzzati, 1953 ▶). This means that there is no sensible way of setting a maximum acceptable level. Note that this is a difference between an *R* factor on intensity, which can be measured as zero or negative, and an *R* factor on amplitude |*F*|, which cannot be negative and will be biased positive if, for example, the *TRUNCATE* procedure of French & Wilson (1978 ▶) has been applied (or indeed any protocol which sets negative values to positive). *R*_merge_ or *R*_meas_ are useful metrics for monitoring variation with batch (§[Sec sec3.1]3.1), and their value for the strongest intensities (top intensity bin or low-resolution bin) is a good indicator of overall data quality. A large *R* factor for the strong data may indicate a problem [a serious cause for concern if *R*_merge_(strong) > 0.10; ideally it should be <0.05], but they are not good indicators for weak data. In general, since *R* factors are dependent on the distribution of the data (see, for example, Murshudov, 2011 ▶) they are not good indicators of model quality or internal consistency, whereas the correlation coefficients are indicators of the degree of linear dependence between data sets and are less dependent on the distribution of the data, so they may be better indicators (see Appendix *A*1[App appa]).

**Figure 2:**
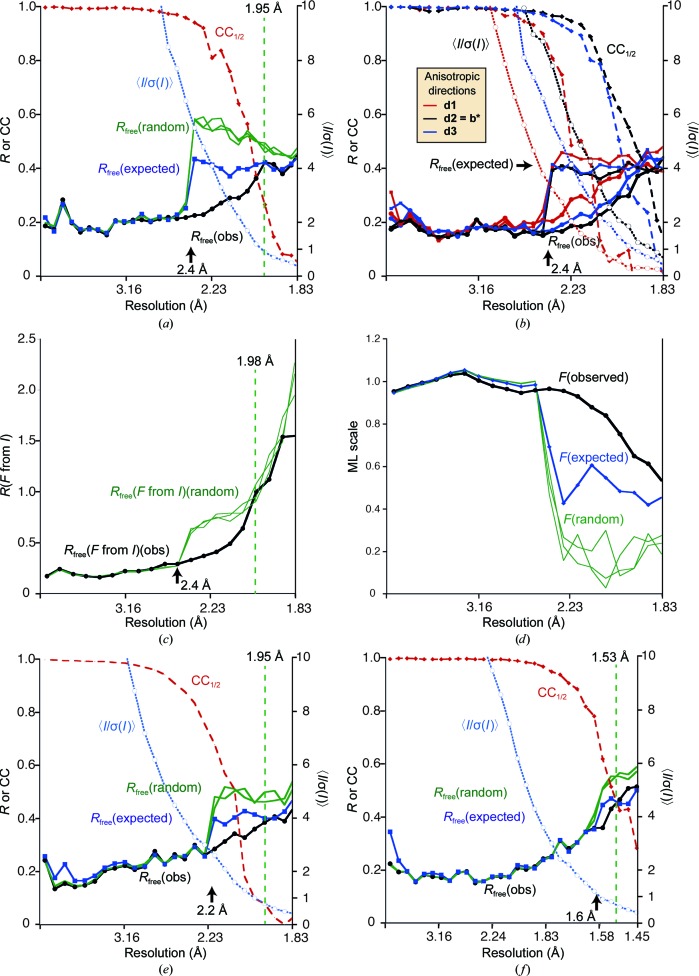
Plots of data-processing and refinement statistics against resolution. In (*a*), (*b*), (*e*) and (*f*), CC_1/2_ is shown as a dashed red line and 〈〈*I*〉/σ(〈*I*〉)〉 is shown as a pale blue dotted line (right-hand axes). (*a*), (*b*), (*c*) and (*d*) are from example 1, (*e*) is from example 4 and (*f*) is from example 5. (*a*) Data-processing statistics and *R*_free_ for the observed data (black); against simulated data beyond 2.4 Å resolution, expected |*F*| (blue); and |*F*| from two or three sets of random intensities (green). (*b*) Similar statistics in cones around the three principal directions of anisotropy **d1** (red), **d2** (= b*, black) and **d3** (blue), omitting the *F*(random) values. (*c*) *R*_free_(*F* from *I*) values for refinement against measured and random simulated intensities. (*d*) ML scale that indicates the contribution of these reflections to electron-density calculations. (*e*) As (*a*) but for example 4. (*f*) The same for example 5

A better measure for assessing the ‘resolution’ of a data set is the correlation coefficient between random half data sets, CC_1/2_ (labelled ‘CC_Imean’ in older versions of *SCALA*; Evans, 2006 ▶, 2011 ▶; Karplus & Diederichs, 2012 ▶). This statistic is plotted against resolution in both *SCALA* and *AIMLESS* (Fig. 2 ▶). A related statistic, Fourier shell correlation, has been used for assessing the resolution of electron-microscopy reconstructions since the early 1980s (see, for example, Rosenthal & Henderson, 2003 ▶; Henderson *et al.*, 2012 ▶). The advantage of a correlation coefficient is that it has a well defined range: +1.0 for a good correlation and 0 for no correlation. CC_1/2_ is generally close to 1 at low resolution and falls sharply to near zero at higher resolution as the intensities become weaker (Fig. 2 ▶).

#### 3.2.3. Anisotropy

Most data sets are anisotropic to some extent, which complicates analysis and decision making. The anisotropy of the data is analysed in *AIMLESS* using both CC_1/2_ and 〈*I*/σ〉. The two or three orthogonal principal directions of anisotropy are constrained by the lattice symmetry: no anisotropy for cubic symmetry, two principal directions, the unique *c* (c*) axis and the *ab* plane, for tetragonal, hexagonal and trigonal, three orthogonal axes for orthorhombic, the unique *b* (b*) axis and two orthogonal axes in the *ac* plane for monoclinic and three general orthogonal axes for the triclinic system. The general directions for triclinic and in the monoclinic *ac* plane are determined from the eigenvectors of a fitted anisotropic scale factor. Anisotropy is then analysed in cones around the principal axes (default semi-angle 20°) and within the same angle of a principal plane, or alternatively as projections onto the two or three principal directions. In the former case, observations are weighted with a cosine weight declining from 1 along the principal direction to 0 at the edge of the region. Plots of CC_1/2_ and 〈*I*/σ〉 then allow assessment of resolution in different directions in the same way as for the overall resolution and with the same difficulties.

## 4. Tests of resolution cutoffs

How can we decide where to apply a resolution cutoff? On the one hand, using high-resolution data which are so weak as to be insignificant will add nothing useful, may add unwanted noise to maps and may lead us to overconfidence in the quality of our model (although the relationship between data quality and model quality is not clear). On the other hand, we do not want to exclude useful data which might aid structure solution and improve the final model. Anisotropy in the data complicates this decision, as it is not clear whether it is better to include data based on the best direction, the worst, or something in between. Anisotropic cutoffs are likely to cause artefacts in map calculation. The problem of anisotropic data and how to deal with them in refinement and map calculation is an open question, and future work needs to address this problem, with the goal of developing clear protocols.

To examine these questions, a number of tests were carried out using data integrated beyond what would normally be considered acceptable using the example data sets listed in Table 1 ▶.

### 4.1. Comparison with simulated data

By comparing refinement against measured data with refinement against simulated data, we can judge the resolution point at which the measured data become no better than simulated data and compare this with the data-processing statistics. Figs. 2 ▶(*a*)–2 ▶(*d*) show various analyses for example 1, for which simulated data were generated beyond 2.4 Å resolution in several ways with simulations based on the observed intensity distribution but not on the structure itself (see Appendix *A*2[App appa] for details of the simulation): (i) the expected value of |*F*| at the resolution and anisotropic position of each reflection, *i.e.* all |*F*|s close together in reciprocal space are the same (denoted *F*_expected_), (ii) a number of data sets with random intensities around the expected intensities with the same distribution as the measured intensities at the same resolution. These simulated intensities were converted to amplitudes (denoted *F*_random_) with the *CCP*4 program *CTRUNCATE* using the same procedure as used for the experimental data (French & Wilson, 1978 ▶).

The model was then refined with *REFMAC* (Murshudov *et al.*, 2011 ▶) to 1.83 Å resolution against the observed data (*F*_obs_) and also against various simulated data sets. As shown in Fig. 2 ▶(*a*), *R*_free_ values (and *R*_work_; not shown) for the experimental data increase with increasing resolution as expected, while against the simulated data there is a sudden increase at 2.4 Å resolution where the refinement switches from the experimental data, but beyond about 1.95 Å resolution *R*_free_ for *F*_obs_ is no better than that for *F*_expected_, while refinement against random data sets (with different errors) gives *R*_free_ values which converge towards the values from *F*_obs_ and *F*_expected_ at higher resolution at around *R* = 0.42, the expected value (see Appendix *A*1[App appa]). We could conclude from this that there is no gain in including data beyond about 1.95 Å resolution, at which point CC_1/2_ is 0.27 and 〈*I*/σ〉 is 0.9: a more conventional cutoff point at 〈*I*/σ〉 = 2.0 would be at 2.06 Å resolution. This data set is significantly anisotropic, with orthogonal principal axes along **d1** = 0.97*h* + 0.23*l*, **d2** = *k*, **d3** = −0.75*h* + 0.67*l*, with the resolutions at which 〈*I*/σ〉 = 2.0 along **d1**, **d2** and **d3** being 2.23, 1.98 and 2.05 Å, respectively (Fig. 2 ▶ *b*). Analysis of *R* factors in cones in the same way (§[Sec sec3.2.3]3.2.3 and Fig. 2 ▶ *b*) shows a similar pattern to the overall values, with a convergence of *R* factors against *F*_obs_ and *F*_expected_ in the range 2.08–1.91 Å.

The *TRUNCATE* procedure for inferring |*F*| from experimentally measured *I* produces a positive bias for weak intensities, which complicates the comparison with simulated data. To test the effect of this, refinement was also carried out against observed and simulated intensities instead of against amplitudes: *R*_free_ values against simulated data were larger from 2.4 Å resolution as before, but had converged by ∼1.98 Å resolution (Fig. 2 ▶ *c*). Following refinement against intensities, *REFMAC* calculates *R* factors in a way that mimics *R* factors on *F* (Murshudov *et al.*, 2011 ▶), but these *R* factors rise sharply with resolution (ultimately to infinity), rather than flattening out as do those on |*F*|.
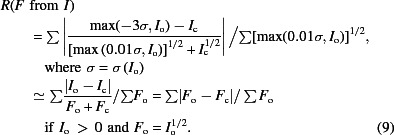



Another estimate of the significance of the data is shown by the maximum-likelihood scale factor *D*. The best electron density after refinement is calculated using coefficients 2*mF*_o_ − *DF*_c_, where *m* is dependent on *D*. The values of *D* within resolution shells indicate how much these reflections contribute to electron density, with a value close to zero indicating that these reflections make little contribution. Fig. 2 ▶(*d*) shows that (for example 1) *D* falls with resolution but is still greater than the values for simulated data even at the resolution edge.

Similar tests were carried out for examples 4 and 5 (see Figs. 2 ▶ *e* and 2 ▶ *f*), with similar conclusions that by the time CC_1/2_ has fallen to around 0.2–0.4, or 〈*I*/σ〉 to around 0.5–1.5, there is little information remaining, but that it would be hard to make a definite rule. With increasing resolution the *R* factors and *D* values for experimental data converge towards the values from simulated data, but the point at which they coalesce relative to the data-processing scores varies between different scores and different data sets.

### 4.2. Tests with automated model building

There have been anecdotal reports that extending the resolution to include weak data may help automated model-building procedures. Unfortunately, this has been hard to prove: the examples tried here either worked at all resolutions or largely failed at all resolutions. Example 2 was used to test model building from a rather poor map with experimental phases to 2.5 Å resolution and model building with the *Buccaneer*/*REFMAC* pipeline (Cowtan, 2012 ▶) tested at resolutions of 2.3, 2.4, 2.5 and 2.6 Å. Models built at all of these resolutions had some correct and some incorrect parts, with the assigned sequence being largely wrong, but there was no consistent pattern over the different resolutions in the number of residues built or sequence assignment or in the correctness of the models. Another test was performed on example 3, a 2:1 complex, starting with a molecular-replacement model with the two large molecules (595 residues each) and building the smaller 91-residue molecule. In this case the smaller component was built more or less consistently at any resolution between 2.57 and 3.3 Å, although perhaps with fewer errors away from these extremes at between 2.7 and 3.1 Å. Similar results were obtained with example 4, omitting the last domain (207 residues): this could be rebuilt more or less successfully at resolutions between 1.8 and 2.8 Å, although maybe slightly more correctly at higher resolutions. However, extending the resolution at least seemed to do no harm. It might indicate that the *Buccaneer*/*REFMAC* pipeline for model building at least is more dependent on phase error rather than quality of structure-factor amplitudes (and hence resolution). In cases of difficulty, it may be worth trying model building with data to different resolutions.

### 4.3. Electron density in OMIT maps

**Figure 3:**
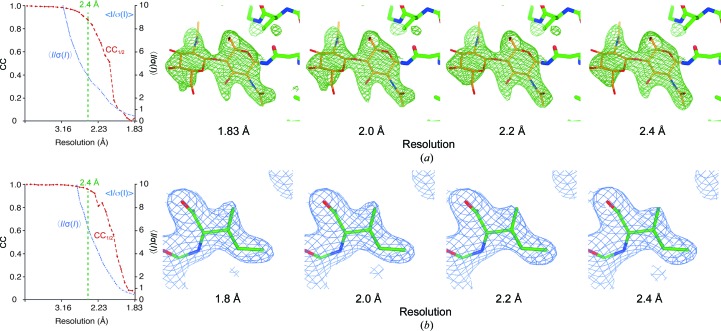
OMIT difference maps (*mF*_o_ − *DF*_c_) at different resolutions, along with data-processing statistics plotted against resolution. (*a*) A sugar (NAG) chain from example 4. (*b*) An omitted residue from example 1.

The effect of resolution on the visual appearance of difference maps was tested on regions of the model which were not included in refinement. This is important in manual building and completion of structures. Part of a structure was omitted, the remaining coordinates were perturbed by a random shift of up to 0.3 Å to reduce model bias and the structure was refined in *REFMAC* with different resolution cutoffs. Fig. 3 ▶ shows two examples of maximum-likelihood difference maps at different resolutions for examples 4 and 1. There is not much difference between the maps, although there may be a slight improvement in sharpness in example 1 (Fig. 3 ▶ *b*) on extending from 2.4 to 2.0 Å resolution, consistent with the refinement results in §[Sec sec4.1]4.1 (Fig. 2 ▶ *a*). Again, as for the automated model building (§[Sec sec4.2]4.2), extending the resolution seems at least to do no harm.

## 5. Discussion

The program *AIMLESS* performs essentially the same task as *SCALA* and gives similar results. However, it is significantly faster (about three times) and is a better framework for adding new scaling models and analyses. In due course, the three programs *POINTLESS*, *AIMLESS* and *CTRUNCATE* may be combined into one.

The extensive statistics produced by *AIMLESS* are mostly similar to those produced by *SCALA*, so the questions and decisions for the user are as discussed in Evans (2011 ▶): (i) What is the point group (Laue group)? (ii) What is the space group? (iii) Is there radiation damage and should the most damaged regions of data be excluded? (iv) What resolution cutoff should be applied (see below)? (v) Is there a detectable anomalous signal? (vi) Are the data twinned? (vii) Is this data set better than those previously collected?

With regard to this last point, one traditional way of improving weak or incomplete data is to merge data from different crystals, provided that they are isomorphous. With the advent of cryocooling, this has fallen out of fashion except for the most desperate cases, but recent work from Hendrickson’s group (Liu *et al.*, 2012 ▶, 2013 ▶) makes a good case for merging data from many crystals to enhance the very weak anomalous signal from sulfur. The current tools for checking isomorphism between crystals are rather undeveloped, but this technology is improving (see Giordano *et al.*, 2012 ▶). With the fast data collection on modern synchrotrons, it is common to collect several or many more-or-less equivalent data sets and merging them may be better than just choosing the best. At the end, model quality depends on data quality and merging many data sets usually improves the signal-to-noise ratio in the data; however, it is not clear how to merge data when there is severe non-isomorphism or radiation damage. Blindly merging data may do more harm than good. It is necessary to analyse the joint distribution of all data sets and to merge using this distribution. In an ideal world all data sets would be used without merging, thus ensuring that the extraction of information from the data would be optimal at all stages of structure analysis: the distribution of the data and the amount of information available at each stage of analysis would define what needs to be used.

A major cause of user indecision and conflicts with journal referees is the resolution cutoff. We cannot set definite rules for this, as it depends on what the data are to be used for. It is therefore a mistake to prematurely apply a harsh cutoff at the data-reduction stage: data can always be excluded later. It is also unclear how best to handle anisotropy: do you choose the best direction or the worst? It is probably best to include data to the limit in the best direction. Tests carried out here to relate the resolution statistics to final model building and refinement do suggest that extending the data somewhat beyond the traditional limits such as 〈*I*/σ〉 = 2 may improve structure determination, as do the ‘paired-refinement’ tests of Karplus & Diederichs (2012 ▶). At the very least, adding these weak data seems to do no harm for the purposes of either automatic or manual model building. The main problem is that we have become accustomed to using the nominal resolution as an indicator of model quality, and it is not a good indicator, particularly as important biological and chemical conclusions from a structure often depend on local rather than global correctness. Nor indeed can any global score can indicate the correctness of any local structural feature. Clearly, well measured data to 1.5 Å resolution contain more information than a data set to 3.5 Å resolution and are therefore likely to lead to a more correct structure, but nominal resolution in itself just tells us how many reflections were used, rather than their quality. From our limited tests here, it seems that changing the resolution cutoff over a considerable range (*e.g.* from 2.2 to 1.9 Å) makes only a small difference, so the exact cutoff point is not a question to agonize over, but it seems sensible to set a generous limit so as not to exclude data containing real (if weak) information. There is no reason to suppose that cutting back the resolution of the data will improve the model. These tests were performed with current programs and our current procedures at all stages could be improved to extract the maximum information from weak noisy data.

## Figures and Tables

**Table 1:** Details of the example data sets used in the tests ‘Resolution’ is the maximum resolution used for integration.

Example	PDB code	Resolution (Å)	No. of residues	Space group	Unit-cell parameters (Å, °)
1	3zym	1.83	3 × 310	*C*2	*a* = 161.1, *b* = 100.3, *c* = 104.0, β = 118.9
2	Unpublished	2.3	211	*P*4_1_2_1_2	*a* = *b* = 60.8, *c* = 144.1
3	Unpublished	2.57	2 × 595 + 91	*P*4_3_2_1_2	*a* = *b* = 128.8, *c* = 267.4
4	3zr5	1.8	656 + 7 NAG	*R*32:*H*	*a* = *b* = 249.9, *c* = 77.8, γ = 120
5	3zyl	1.45	2 × 271	*C*2	*a* = 95.6, *b* = 121.1, *c* = 62.5, β = 110.7

## Footnotes

1 For the naming of the programs *POINTLESS* and *AIMLESS*, see Gibbons (1932 ▶).

2 Note that (as pointed out by a referee) this is only strictly true if all weights used in the calculation of 〈*I*_**h**_〉 are equal: this needs further investigation, although some preliminary tests using different weighting schemes (including equal unit weights) showed only small differences.

## Appendix A Statistical methods

### A1. Behaviour of crystallographic *R* values

Crystallographic *R* factors are calculated using the formula

where the summation is over the reflections used to calculate the *R* value (in the case of twinning a generalization of this formula is used; see, for example, Murshudov, 2011 ▶). Obviously, the behaviour of this statistic depends on the statistical properties of the structure factors. Therefore, it can be expected that properties of the model, crystal and observed data such as (i) noisy data, (ii) twinning, (iii) modulation in crystals and (iv) model errors will affect the behaviour of the *R* value. Luzzati (1953 ▶) analysed the effect of model errors on *R* values in the absence of any other peculiarity and came to the conclusion that *R* values calculated for structure factors calculated from random atoms would be around 0.58 for acentric reflections. Murshudov (2011 ▶) carried out a similar analysis for cases of hemihedral twinning and demonstrated that *R* values for cases of hemihedral twinning are systematically lower than those for single crystals. Here, we analyse the effect of the procedure used to estimate the amplitude |*F*| from the measured intensity *I* when data are very noisy. Since weak intensities may be measured as negative, while the true amplitude |*F*| cannot be negative, |*F*| is generally estimated using the *TRUNCATE* procedure (French & Wilson, 1978 ▶). Under the assumption that true structure factors come from a crystal filled with atoms randomly distributed over the unit cell and the noise in the experimental intensities has a normal distribution, the *TRUNCATE* procedure estimates the amplitudes of structure factors using a Bayesian estimation with the formula

where *P*(*J*; *I*_o_, crystal) is the conditional distribution of ideal intensities of structure factors (*J*) when observed intensities (*I*_o_) are known and it is known that the data came from a crystal. Noting that |*F*| = *J* ^1/2^, and using the explicit form of *P*(*J*; *I*_o_, crystal), the conditional distribution for |*F*| can be written

where ∊Σ = *E*(|*F*|^2^), the expected value of |*F*|^2^, is estimated using the data in the resolution bins, *I*_o_ is the experimental observed intensity and σ is its standard deviation. Thus, the expected values of the amplitudes of structure factors are estimated using the formula

Although this formulation has served the community well over the years, it has certain problems. These problems include the following. (i) It is assumed that Σ is a smooth function of resolution, which breaks down in the presence of pseudo-translation, DNA/RNA helices *etc.* (ii) It is assumed that the data are from single crystals. In cases of twinning the formulation may not work, although if the twinning fraction is known and there is no noncrystallographic rotation parallel to the twin operators then it is straightforward to account for twinning. However, in general not all properties of the crystal/data are known or possible to model. (iii) It is assumed that the observed data have a normal distribution. This assumption may break down, especially for weak reflections where profiles of neighbourhood spots are used for integration.

When the data are very noisy (*i.e.* the standard deviation of the observation becomes very large) this procedure produces the expected value of Wilson’s distribution (Wilson, 1949 ▶),

Thus, in the limiting case if all estimation of parameters proceeds smoothly and there are no crystal-growth peculi­arities, then the *TRUNCATE* procedure would give the expected value of the Wilson distribution. It is interesting to analyse the crystallographic *R* values for these cases. Let us assume that the *R* value is calculated in the resolution bins where Σ was estimated. Then, in the limiting case of noisy data the *R* value would have the following form (here, we assume that reciprocal-space points are sufficiently dense and the summation can be replaced by the integration),

Now if we use the fact that the distribution of |*F*_c_| is the same as the distribution of |*F*|, that is the Wilson distribution, and denote μ = *E*(|*F*|) = *E*(|*F*_c_|), then after some manipulation of integrals we can derive

where erfc is the complementary error function,

Thus, we see that if structure factors are replaced by the expected value of the Wilson distribution then it can be expected that the calculated *R* values will be around 0.42. This is exactly what happens with observed values from the *TRUNCATE* procedure when errors in the experimental intensities become very large. It should be noted that this behaviour of *R* values is a property of data from single crystals with no other peculiarities.

Note that for perfect hemihedral twinning

If the perfect twinning assumption is used in the *TRUNCATE* procedure then for very noisy data we can obtain

### A2. Generation of random intensities from Gaussian χ^2^ distribution

Under the assumption that the ‘true’ structure factors came from the population with a Wilson distribution and experimental intensities have a normal distribution with mean value equal to the ‘true’ intensities and with experimental uncertainties equal to σ, the distribution of observed intensities can be written

To use this formula for random intensity generation it is necessary to estimate the unknown parameters (Σ). If we want to generate ‘data’ beyond the resolution of the observed data we need to parameterize Σ as a smooth function of resolution. We use the parameterization

where *k* is the overall scale, *B* is the overall isotropic temperature factor, *f*(*s*) is the empirical intensity curve designed by Popov & Bourenkov (2003 ▶), *s* is a reciprocal-space vector, |*s*| is the length of the reciprocal-space vector and *U* is the overall anisotropic *U* value with properties

where tr is the trace, *R*_sym_ is the rotation part of a symmetry operator (here, we use the orthogonal version of the symmetry operators) and the superscript *T* denotes the transpose of the operator. The second condition in (22) is for all symmetry operators of the crystal. Since (21) is a continuous function of Σ, it can be used for any resolution including resolutions beyond the observed resolution.

The procedure for estimation of parameters and generation of random intensities is as follows.

(i) Using *E*(*I*_o_) = ∊Σ, var(*I*_o_) = (∊Σ)^2^, build a Gaussian approximation for the distribution of the observed intensities and estimate parameters of Σ as defined by (21).
(ii) Using the distribution (20), build the likelihood function and improve the estimation of the parameters of Σ using maximum-likelihood estimation.
(iii) Generate expected values of the amplitudes of structure factors for reflections within defined resolution using the formula *E*(|*F*|) = (π∊Σ)^1/2^/2.
(iv) Generate random intensity from the population defined by the distribution (20). For this, a two-stage procedure is used: (*a*) generate a random number from the exponential distribution with mean ∊Σ and denote it *I*_exp_ and (*b*) generate a random number from the normal distribution with mean *I*_exp_ and standard deviation σ. Σ is extrapolated to the given resolution and σ is taken from the observed data or from the defined signal-to-noise ratio.
(v) Generate random numbers from the population with Wilson distribution with the parameter ∊Σ.
